# Assessment of the Influence of Environmental Conditions on the Corrosion Rate of Reinforcing Bars in Concrete Made with Two Different Cements †

**DOI:** 10.3390/ma19183998

**Published:** 2026-09-20

**Authors:** Wioletta Raczkiewicz, Kamil Bacharz, Albert Grajek

**Affiliations:** Faculty of Civil Engineering and Architecture, Kielce University of Technology, Al. 1000-lecia PP 7, 25-314 Kielce, Poland; kbacharz@tu.kielce.pl (K.B.); grajekalbert@hotmail.com (A.G.)

**Keywords:** RC specimens, blast furnace slag cement, reinforcement corrosions, galvanostatic pulse method, chloride ions, freeze and thaw cycles

## Abstract

In recent years, low-carbon concretes have undergone intensive development in response to the need to reduce CO_2_ emissions, particularly in the construction sector. One of the approaches to reducing the carbon footprint is the use of blended cements, including blast furnace cement CEM III containing a significant proportion of ground granulated blast furnace slag and composite cement CEM V. The aim of the study was to evaluate the effect of CEM III and composite cement CEM V on reinforcement corrosion and selected properties of structural concrete. Two concrete mixtures differing only in cement type were prepared. The experimental program included the evaluation of reinforcement corrosion activity using the Galvanostatic Pulse Method (GPM) on reinforced concrete specimens exposed to different environmental conditions. Additional tests included compressive strength, modulus of elasticity, shrinkage, freeze–thaw resistance, and water absorption. Based on the results obtained, it was concluded that, under the investigated conditions, namely exposure of reinforced concrete specimens to chloride ions and freeze–thaw cycles, CEM V cement may constitute an alternative to CEM III cement. This is particularly relevant because the potential environmental benefits associated with the lower CO_2_ emissions of concretes containing CEM V cement are supported by the available literature. Nevertheless, to verify this assumption, more detailed studies on the durability of reinforced concrete structures are required.

## 1. Introduction

The construction industry is one of the key sectors of the global economy. Urbanization, industrialization, and the continuous development of transport infrastructure require the ongoing production of construction materials, particularly concrete and steel. Currently, the construction sector faces the challenge of significantly reducing carbon dioxide emissions in line with the objectives of the European Green Deal [1]. Achieving sustainability in the construction industry is particularly challenging due to its highly industrialized nature and extensive consumption of raw materials and manufactured products. Among its branches, the cement industry is one of the most carbon-intensive sectors, with cement production accounting for approximately 6–8% of global CO_2_ emissions [2].

The production of ordinary Portland cement with a high clinker content Is the most carbon-intensive process [3]. Therefore, replacing a portion of the clinker with additional cementitious materials can significantly reduce CO_2_ emissions associated with cement production [4,5,6]. At the same time, this approach allows the production of concrete with a lower carbon footprint [7]. Consequently, considerable research efforts are currently focused on the development of low-carbon cements. The authors of [8], who investigated CEM II/C-M (S-V), CEM VI (S-V), CEM III/A 42.5N, and CEM II/C-M (S-LL) cements, reported that the use of composite cements instead of ordinary Portland cement can reduce CO_2_ emissions to approximately 340–450 kg per tonne of cement. In another study [9], new composite cements were developed in which 40–55% of Portland clinker was replaced with a mixture of limestone, ground granulated blast furnace slag and fly ash. The authors concluded that these cements should be further developed and implemented in construction practice, as they enable a substantial reduction in CO_2_ emissions while maintaining the required performance characteristics of concrete. Promising research on reducing the clinker content of next-generation cements was presented in [10], where calcined clays were identified as an effective supplementary cementitious material for partially replacing Portland clinker.

Blast furnace cement (CEM III) is an effective alternative to ordinary Portland cement, particularly in structural concrete applications. This type of cement contains ground granulated blast furnace slag (GGBFS), which partially replaces Portland clinker. It is characterized by a low heat of hydration and high resistance to aggressive environments, including chloride ions, sulphates, and groundwater. In addition, blast furnace cement improves the density of the concrete microstructure, reduces porosity, and provides excellent long-term strength development [11].

Another promising type of cement is composite cement (CEM V), in which Portland clinker is partially replaced by ground granulated blast furnace slag and calcareous or siliceous fly ash. This cement is characterized by a low heat of hydration, high durability, and excellent resistance to chemical attack [12]. It also exhibits a dense, fine-pored microstructure and good resistance to frost [13]. However, due to its slower setting and lower early-age strength development, CEM V is generally not recommended for precast concrete production [14]. Furthermore, the study reported in [15] demonstrated that concrete made with CEM V cement is more susceptible to carbonation, with carbonation depths that may be significantly greater than those observed in concrete produced with ordinary Portland cement (CEM I) without mineral additions.

When considering concrete as a structural material, it should be emphasized that, in addition to providing adequate compressive strength, one of its primary functions is to protect the reinforcing steel against corrosion, thus ensuring the durability of reinforced concrete structures. Progressive corrosion may lead to a reduction in the cross-section of reinforcing steel, cracking and deterioration of the concrete cover, and degradation of the steel–concrete interaction, which may ultimately reduce the load-bearing capacity and reliability of reinforced concrete structures. In particular, chloride-induced corrosion is an important factor in the time-dependent reliability and long-term safety assessment of reinforced concrete structures [16]. Numerical and electrochemical approaches are also increasingly used to assess the development and spatial distribution of reinforcement corrosion under different exposure and electrochemical conditions [17].

Depending on the class of structural design and the environmental exposure class specified by the applicable standards, an appropriate class of concrete strength and the minimum cement content must be selected in accordance with the relevant requirements [18]. To date, ordinary Portland cement has been widely used in structural concrete because it provides high resistance to carbonation [19,20], although it is not the most suitable choice for structures exposed to aggressive environments. For structures subjected to chloride- or sulfate-induced attack, the use of blast furnace cement (CEM III) containing ground granulated blast furnace slag is recommended [21,22]. The hydration and hardening processes of concretes made with CEM III proceed more slowly, and their lower heat of hydration results in reduced internal stresses. Consequently, the shrinkage strains are lower and develop more gradually, effectively limiting the penetration of aggressive substances into the concrete microstructure.

Currently, the need to reduce CO_2_ emissions has made it necessary to replace ordinary Portland cement with cements containing supplementary cementitious materials. Although extensive research has been conducted on blended cements and concretes that incorporate such binders (see references cited above), studies investigating their influence on the durability of reinforced concrete structures, particularly the corrosion development of embedded reinforcing steel, remain relatively limited and often provide inconclusive results. In this field, the authors of [23] reviewed studies on the corrosion of reinforcing steel in alkali-activated concretes exposed to chloride-rich environments, such as seawater and de-icing salts. Investigations used half-cell potential (HCP), Linear Polarization Resistance (LPR), and electrochemical impedance spectroscopy (EIS) techniques. The authors concluded that these methods are suitable for assessing the durability of low-carbon concretes. Their review showed that alkali-activated concrete often exhibits greater resistance to chloride intrusion and a longer corrosion initiation period than conventional ordinary Portland cement (OPC) concrete. However, the correlation between laboratory results and reinforced concrete under actual service conditions has not yet been proved conclusively. The study presented in [24] investigated the long-term effect of the addition of fly ash on carbonation and reinforcing steel corrosion in concrete. The research was carried out over a 5-year period under laboratory conditions and, partially, under natural exposure. Concrete mixtures containing different proportions of fly ash were compared with a reference concrete produced exclusively with ordinary Portland cement. The results indicated that fly ash concretes exhibited greater carbonation depths and higher reinforcement corrosion rates under elevated CO_2_ concentrations. However, the authors emphasized that the behaviour of concrete under natural environmental conditions is considerably more complex and does not always correspond to the results obtained from accelerated laboratory tests. The publication [25] reported the influence of low-carbon cementitious binders on the passivation of reinforcing steel. The authors demonstrated that some environmentally friendly binders are capable of providing corrosion protection comparable to, or even better than, that of conventional Portland cement by promoting the formation of a stable passive layer on the reinforcement surface. However, they emphasized that not all low-carbon binders are suitable for reinforced concrete applications and that each material should be individually evaluated with respect to its durability and its ability to protect reinforcing steel against corrosion. The durability of concretes containing cements with a reduced Portland clinker content was also investigated by the authors of [26]. They applied the electrical resistance corrosion monitoring method to evaluate corrosion-related changes in reinforcing steel embedded in low-carbon concretes subjected to carbonation. Their results demonstrated that reducing the content of Portland clinker increases the susceptibility of concrete to carbonation, thus decreasing its ability to protect reinforcing steel against corrosion. The authors also concluded that the durability and protective performance of these concretes depend not only on the clinker content, but also on the reactivity of the additional cementitious materials used.

The authors of [27] investigated the long-term corrosion behaviour of reinforcing steel in low-carbon concretes containing a high proportion of supplementary cementitious materials (SCM), such as fly ash and/or ground granulated blast furnace slag. The specimens were exposed both to NaCl solutions and to a natural marine environment, allowing a comparison between laboratory and field conditions. Corrosion was evaluated using the half-cell Potential (HCP) method. The results indicated that low-carbon concretes with a high content of SCM can delay corrosion initiation; however, in the long term, particularly under marine exposure, corrosion can still develop once the critical chloride threshold at the reinforcement level is exceeded. Furthermore, the authors concluded that laboratory exposure to NaCl solution does not always accurately reproduce the behaviour of reinforced concrete under actual service conditions. An interesting study was presented in [28], where concrete incorporating additional cementitious materials, including silica fume, ground granulated blast furnace slag, and fly ash, was investigated at cement replacement levels ranging from 10% to 60%. The results demonstrated that adequately selected replacement ratios, particularly 10% replacement with silica fume, improved compressive strength, reduced chloride and sulphate ingress, and decreased the susceptibility of reinforced steel to corrosion. In studies [29,30], the corrosion behaviour of AISI 316 and AISI 1018 steels embedded in green concrete was investigated. The study [29] examined the effects of incorporating sugarcane bagasse ash (SCBA) and silica fume, while the study [30] evaluated the combined use of sugarcane bagasse ash and recycled aggregates. The corrosion performance was evaluated using electrochemical techniques based on measurements of the corrosion potential and the corrosion rate during exposure to seawater. The results showed that the partial replacement of Portland cement with pozzolanic materials improved the density of the cementitious matrix and reduced the corrosion of stainless steel. In contrast, the use of supplementary materials and recycled aggregates produced more variable results. Some studies have focused on the use of alternative binders as substitutes for conventional Portland cement and their influence on the durability of reinforced concrete structures. In recent years, particular attention has been devoted to geopolymer binders. The study reported in [31] investigated the chloride-induced corrosion resistance of reinforcing steel embedded in geopolymer concrete based on low-calcium fly ash and ground granulated blast furnace slag. The results demonstrated that geopolymer binders can effectively limit corrosion development because of their low chloride-ion permeability. In another study [32], the corrosion behaviour of reinforcing steel embedded in geopolymer concretes produced with low-calcium and high-calcium fly ashes was compared. The experimental program included 450 days of accelerated carbonation followed by electrochemical measurements to evaluate reinforcement corrosion. The authors demonstrated that low-calcium geopolymer binders provide significantly better corrosion protection than high-calcium geopolymers. However, the practical application of geopolymer binders is still limited by their relatively high production costs, the insufficient knowledge regarding their long-term durability, and the need for individual verification of their performance characteristics.

In the case of reinforcing steel corrosion in low-carbon cements, understanding the rate of corrosion development under the combined action of chloride ions and freeze–thaw cycles is of particular importance. However, only a limited number of studies have addressed this issue. The study reported in [33] investigated the effects of fly ash, straw fibres, and rubber granules on freeze–thaw degradation and chloride-induced corrosion resistance of alkali-activated slag-based cementitious materials (AASCMs), considered a low-carbon alternative to ordinary Portland cement. The incorporation of appropriately selected modifiers improved the compactness of the material. The authors concluded that low-carbon cementitious materials can achieve satisfactory durability, provided that their composition is optimized for exposure to freezing conditions and de-icing salts. The study presented in [34] examined chloride migration in concretes with different degrees of freeze–thaw deterioration, both with and without supplementary cementitious materials (SCM). The results demonstrated that repeated freeze–thaw cycles promote microcrack formation and increase water absorption, thus significantly accelerating chloride ingress. Air-entrained concrete exhibited the highest frost resistance, while concrete containing ground granulated blast furnace slag (GGBFS) showed high resistance to chloride migration and good freeze–thaw durability. The authors concluded that proper curing and appropriate selection of SCM can substantially improve the durability of concrete structures exposed to cold climates. In [35], the authors reported the results of a five-year study on the corrosion of reinforced steel embedded in concretes containing additional cementitious materials (SCMs), including fly ash, silica fume, and ground granulated blast furnace slag. The specimens were exposed to a sodium chloride (NaCl) solution that simulates the chloride attack associated with de-icing salts or marine environments. The results showed that concrete incorporating Class C fly ash provided the highest resistance to chloride-induced reinforcement corrosion, while mixtures containing silica fume also exhibited excellent performance. The incorporation of additional cementitious materials improved the density of the cementitious matrix and reduced the rate of chloride ingress into the reinforcement. The authors concluded that properly designed composite cements can significantly improve the durability of reinforced concrete structures exposed to aggressive chloride environments, even when the concrete cover is relatively thin. The literature review indicates that numerous studies have focused on concretes that incorporate blended cements and additional cementitious materials. Several publications have also investigated reinforcement corrosion induced by chloride intrusion or carbonation in such concretes. However, only a limited number of studies have addressed the combined effects of chloride exposure and freeze–thaw cycles on reinforcement corrosion in structural concretes produced with blended cements. Considering the increasing demand for low-carbon cements in modern concrete technology, research in this area is of particular importance.

This article presents preliminary results from a long-term research program aimed at investigating the effect of using composite cement (CEM V) on the corrosion rate of reinforcing steel in concrete and on selected concrete properties under various environmental conditions, including simultaneous exposure to chloride ions and freeze–thaw cycles, an aspect that has rarely been analyzed. The galvanostatic pulse method (GPM) was used to assess the reinforcement corrosion process, as it yields more reliable results than the commonly used half-cell potential measurement method. The properties of concrete made with CEM V cement were evaluated in comparison to concrete made with blast furnace slag cement (CEM III), which is currently widely used in reinforced concrete structures. The observed trends will continue to be monitored and verified in subsequent stages of the research program.

## 2. Materials and Methods

### 2.1. Research Materials

The experimental study was carried out using specimens prepared from two concrete mixtures that differed only in the type of cement used. The first mixture, designated Concrete A, was produced with blast furnace cement CEM III/A 42.5 N MSR NA, while the second mixture, designated Concrete B, was prepared using composite cement CEM V/A (S-V) 42.5N-LH/HSR/NA.

Both mixtures contained basalt aggregate with particle size fractions of 2–8 mm and 8–16 mm (coarse aggregate) and natural sand with a particle size of 0–2 mm (fine aggregate). Tap water that met the requirements of the relevant standards was used for the mixing of concrete. To improve the workability and rheological properties of fresh concrete while reducing the water demand, the high-range water-reducing admixture MasterGlenium ACE 430 TR was added at 0.4% by mass of cement. To ensure the required frost resistance and adequate air entrainment, MasterAir 107 was incorporated at 0.3% by mass of cement. The composition of the concrete mixtures is presented in Table 1.

**Figure 1:**
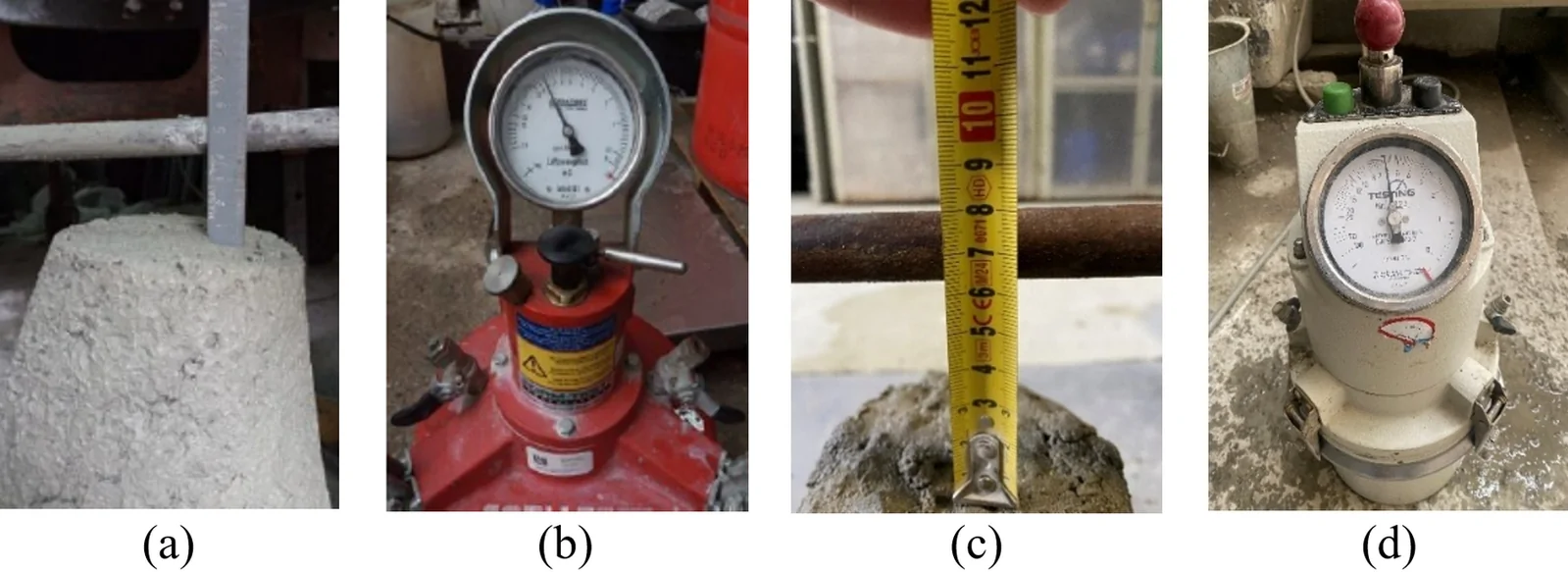
Determination of concrete consistency and measurement of air content in fresh concrete mixtures: (**a**,**b**)—Concrete A, (**c**,**d**)—Concrete B.

Specimens for comparative testing were prepared from both concrete mixtures. All specimens were cast under identical laboratory conditions at a temperature of 20 ± 2 °C and a relative humidity of 50 ± 5%. The consistency of the fresh concrete mixtures was determined using the slump test according to EN 12350-2 [36]. For both mixtures, the measured slump ranged from 5 to 9 cm, corresponding to consistency class S2. The air content of the fresh concrete was determined using a pressure air meter in accordance with EN 12350-7 [37]. In both cases, the measured air content was 6%. Photographs illustrating the measurements of consistency and air content for both concrete mixtures are presented in Figure 1.

According to EN 12390-2 [38], the specimens were demolded 24 h after casting and cured in water for 7 days. Subsequently, they were stored under laboratory conditions for 21 days at a temperature of 20 ± 2 °C and a relative humidity of 50 ± 5%.

### 2.2. Experimental Program and Test Methods

The experimental program involved testing specimens prepared from two concrete mixtures that differ only in the type of cement used. The program included electrochemical measurements to assess the corrosion risk of reinforcing steel in concrete, as well as tests to evaluate selected mechanical and material properties of concrete specimens.

#### 2.2.1. Evaluation of Corrosion Risk and Corrosion Rate of Reinforcing Steel Bars

For the assessment of the corrosion risk of reinforcing steel, twelve prismatic specimens were prepared for each concrete mixture (Concrete A and Concrete B), resulting in a total of 24 specimens. The specimens measured 100 × 100 × 280 mm.

Each specimen contained a single BST500 reinforcing steel bar with a diameter of 8 mm, embedded at a concrete cover depth of 25 mm from the top surface. The reinforcing bars were supplied by the steel manufacturer and were not subjected to grinding, pickling, abrasive blasting, or any other special surface treatment. This approach was adopted intentionally, as one of the research program’s objectives was to replicate as faithfully as possible the actual condition of reinforcing steel used in real reinforced concrete structures.

The ends of the reinforcing bar protruded approximately 10 mm beyond the side faces of the specimen to allow connection to the GalvaPulse measuring device. The specimens were divided into four series, each consisting of three specimens. Each series was exposed to different environmental conditions:

(a) Series I—reference specimens (No. 1–3), stored under laboratory conditions at a temperature of 20 ± 2 °C and a relative humidity of 50 ± 5%;

(b) Series II—specimens (No. 4–6), fully immersed in a 3% NaCl solution and stored under laboratory conditions at a temperature of 20 ± 2 °C and a relative humidity of 50 ± 5% for 37 days;

(c) Series III—specimens (No. 7–9), fully immersed in a 3% NaCl solution and subjected to 100 freeze–thaw cycles in a climatic chamber;

(d) Series IV—specimens (No. 10–12), half-immersed in a 3% NaCl solution and subjected to 100 freeze–thaw cycles in a climatic chamber, with the reinforcing steel bar remaining above the solution level.

**Figure 2:**
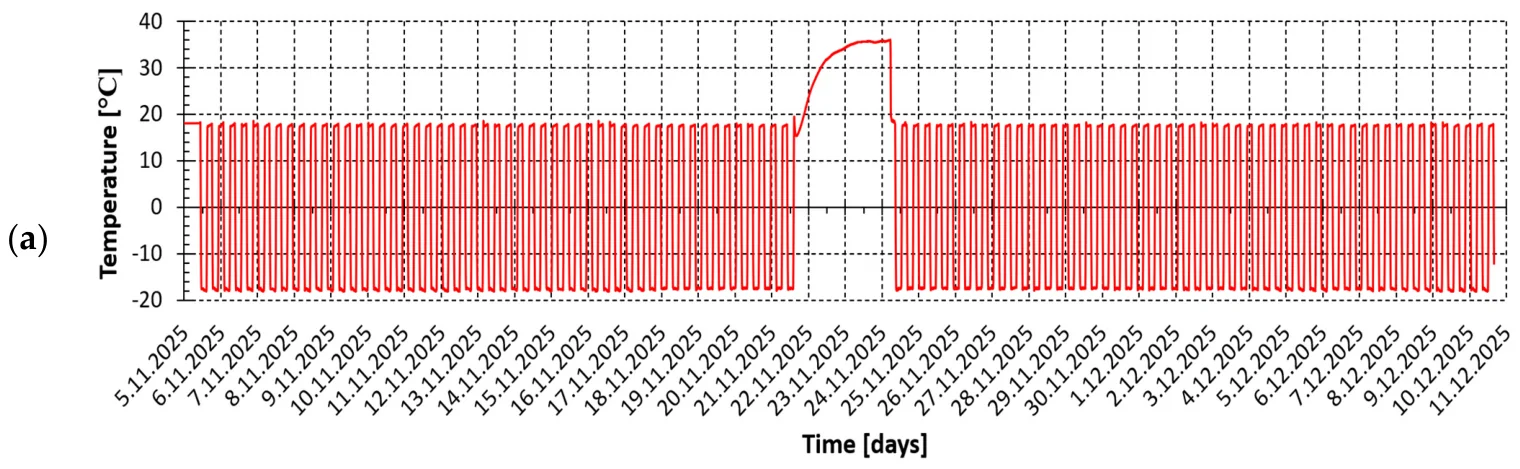

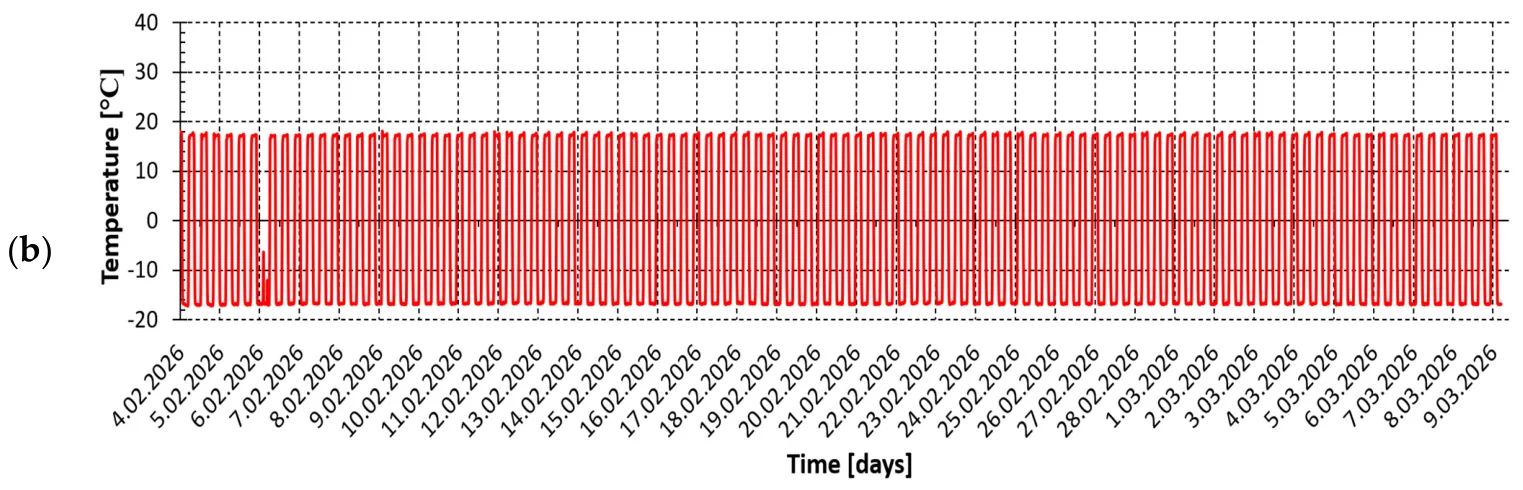
Temperature profiles of the heating and cooling cycles applied to (**a**) Concrete A specimens and (**b**) Concrete B specimens.

For all specimens exposed to the NaCl solution, the protruding ends of the reinforcing bars were sealed with insulating tape and silicone sealant to prevent corrosion outside the concrete. The specimens subjected to freeze–thaw cycles were placed in a climatic chamber operating under an automatically controlled test program. The temperature ranged from +18 °C to −18 °C, resulting in approximately three freeze–thaw cycles per day (Figure 2).

**Figure 3:**
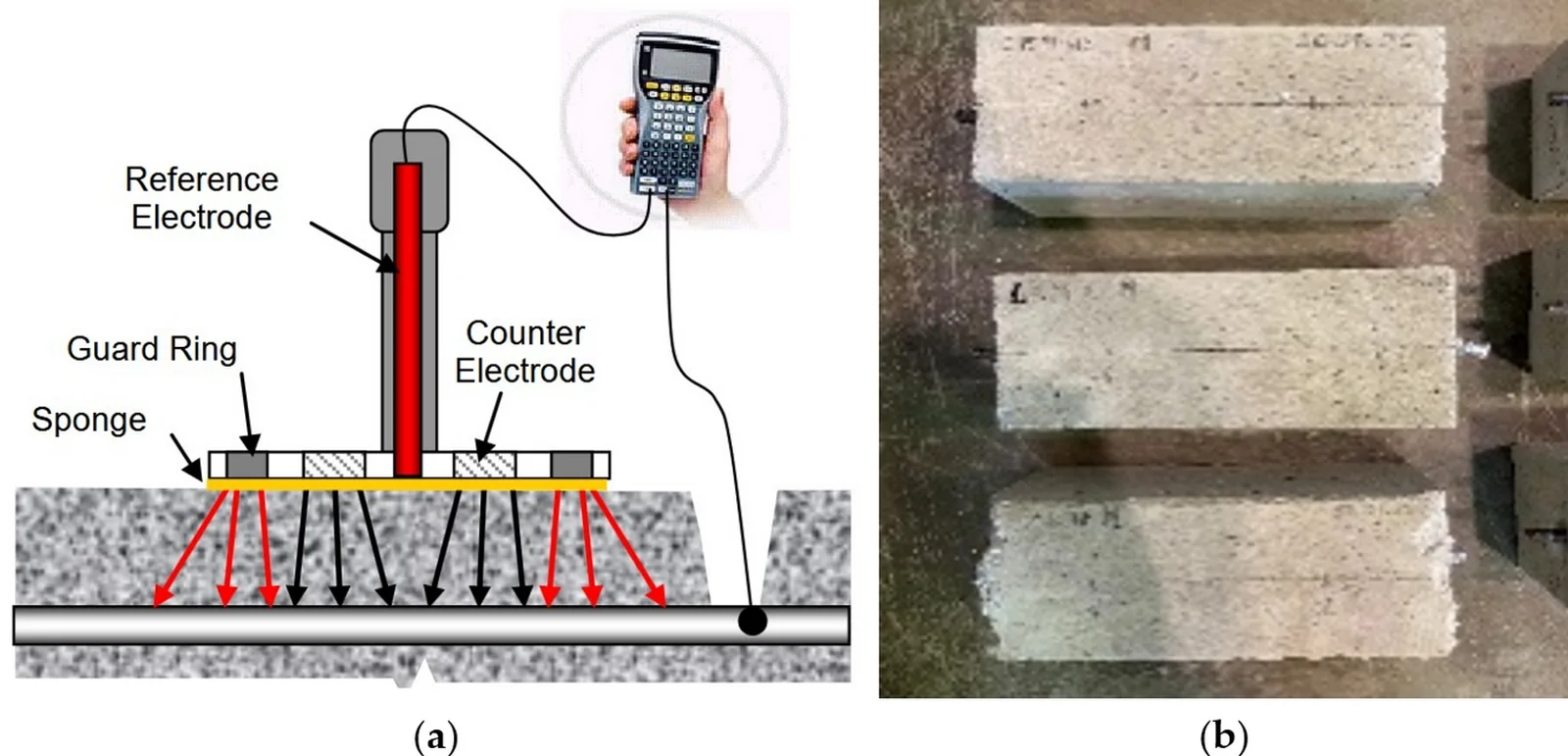
Galvanostatic Pulse Method (GPM): (**a**) schematic illustration of the measurement principle [40], (**b**) representative test specimens.

The corrosion risk and the corrosion rate of the reinforcing steel bars were evaluated using the electrochemical galvanostatic pulse method (GPM) [39] (Figure 3). For each specimen, measurements were made at two locations on the top surface directly above the embedded reinforcing bar. The measurements were carried out in two stages. Stage I comprised the reference measurements, which were performed 29 days after casting, corresponding to the age at which the concrete specimens had reached their standard compressive strength and, under actual construction conditions, could have been exposed to environmental factors shortly after placement. Although hydration processes were still ongoing, both types of concrete were tested at the same age and under identical conditions, enabling a comparison of their behaviour during the early stages of hardening. Stage II involved measurements taken after 100 freeze–thaw cycles of specimens immersed in a 3% NaCl solution, as well as after a corresponding 37-day period of full immersion in a 3% NaCl solution. During each stage, three parameters indicative of reinforcement corrosion were determined: the corrosion current density, which reflects the corrosion activity and corrosion rate of the reinforcing steel, as well as the stationary potential of the reinforcement and the concrete cover resistivity, both of which indicate the probability of corrosion at the measurement location.

Subsequently, the measured values were compared with the threshold values presented in Table 2.

#### 2.2.2. Compressive Strength and Modulus of Elasticity Testing of Concrete

To determine the compressive strength of concrete, fifteen cube specimens measuring 150 × 150 × 150 mm were prepared from each concrete mixture, resulting in a total of 30 specimens. Furthermore, three cylindrical specimens measuring 150 × 300 mm were prepared from each mixture (six specimens in total) for the determination of Young’s modulus using Method B. Uniaxial compression tests were performed according to EN 12390-3:2019 [41], while Young’s modulus was determined in accordance with EN 12390-13 [42]. All tests were carried out using a ZwickRoell Z6000 testing machine (ZwickRoell GmbH & Co., KG, Ulm, Germany) with a maximum compressive load capacity of 6000 kN.

#### 2.2.3. Concrete Shrinkage Testing

**Figure 4:**
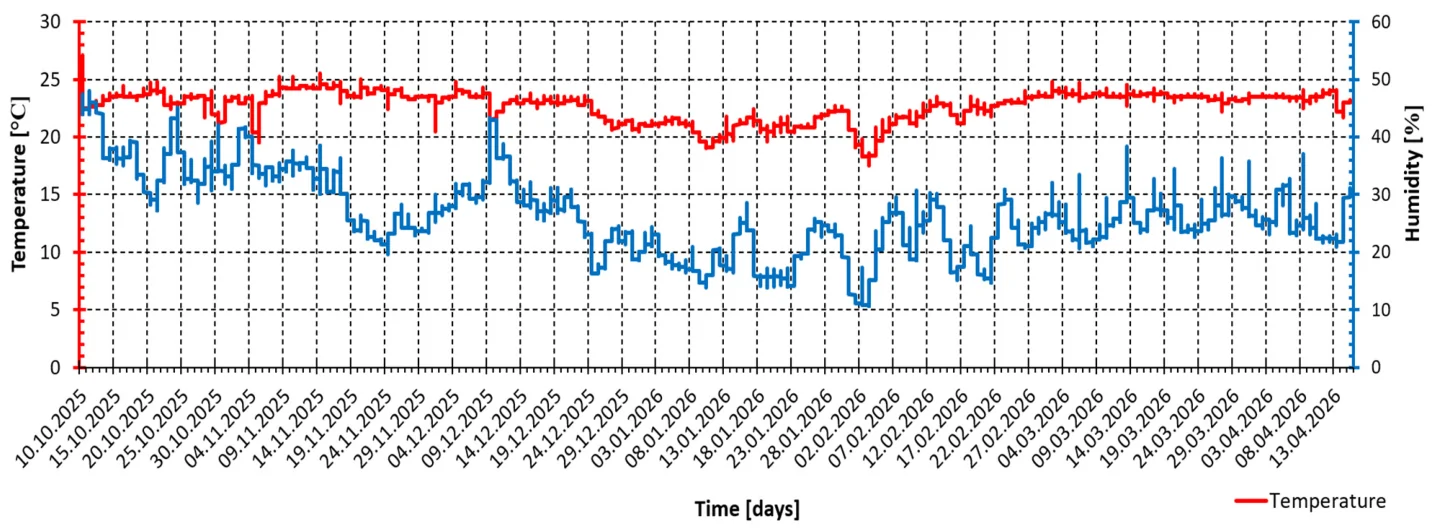
Variations in ambient temperature and relative humidity during concrete shrinkage measurements.

The shrinkage was determined according to EN 12390-16:2019 [43] using prismatic specimens measuring 100 × 100 × 500 mm. Five specimens were prepared from each concrete mixture, resulting in a total of 10 specimens. During the test period, the specimens were stored under laboratory conditions with monitored temperature and relative humidity. The ambient temperature ranged from 20 to 25 °C, with a temporary minimum of approximately 17 °C, while the relative humidity varied between 10% and 49% (Figure 4).

**Figure 5:**
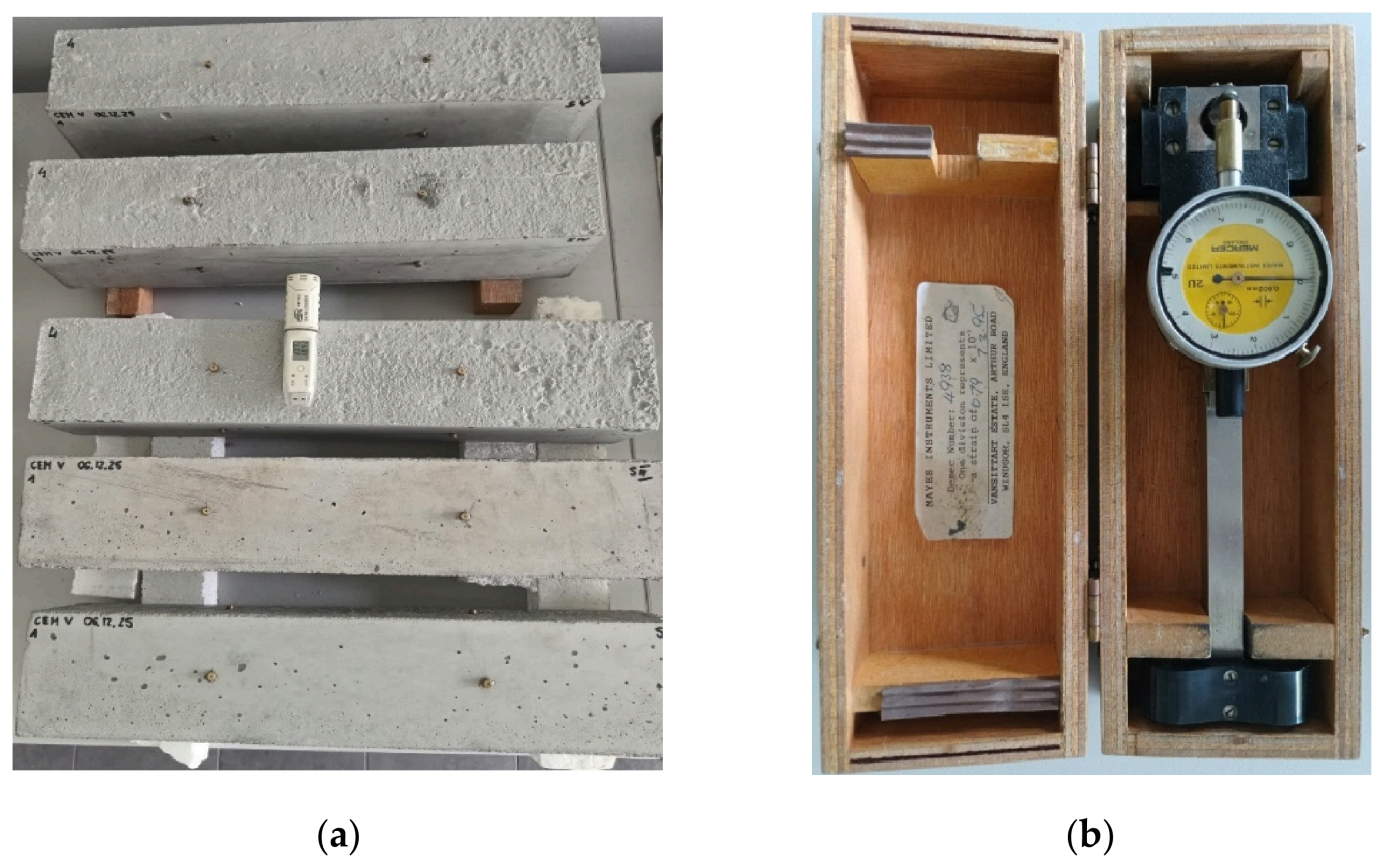
Concrete shrinkage testing: (**a**) representative test specimens, (**b**) Demec mechanical strain gauge used for shrinkage measurements.

Shrinkage was measured on all four lateral faces of each specimen. The measurement gauge length was defined by two metal reference points positioned at one-third of the height of the specimen and spaced as shown in Figure 5a. Measurements were performed using a Demec mechanical strain gauge (No. 4938), manufactured by W.H. Mayes & Son, Windsor, UK (Figure 5b), with a gauge length of 200 mm and a resolution of 0.002 mm; the corresponding gauge constant was 0.79 × 10^−5^. Measurements were taken at predetermined time intervals according to the guidelines provided in the instrument manual [44]: daily during the first 10 days, every 4 days from day 10 to day 26, and every 10 days thereafter.

#### 2.2.4. Freeze–Thaw Resistance Testing

The freeze–thaw resistance of concrete was evaluated using the standard method according to PN-B-06265:2022-08 (Annex N) [45]. For the tests, six cube specimens measuring 100 × 100 × 100 mm were prepared for Concrete A and twelve cube specimens of the same dimensions were prepared for Concrete B, giving a total of 18 specimens. Within each group, half of the specimens were subjected to cyclic freezing and thawing in a freeze–thaw chamber, while the remaining specimens were stored in water and served as reference specimens.

#### 2.2.5. Water Absorption Test

Water absorption was determined according to PN-B-06250:1988 [46] using cube specimens measuring 100 × 100 × 100 mm. Three specimens were prepared for each concrete mixture, resulting in a total of six specimens.

## 3. Results

### 3.1. Results of the Assessment of Corrosion Risk and Corrosion Rate of Reinforcing Steel Bars

**Figure 6:**
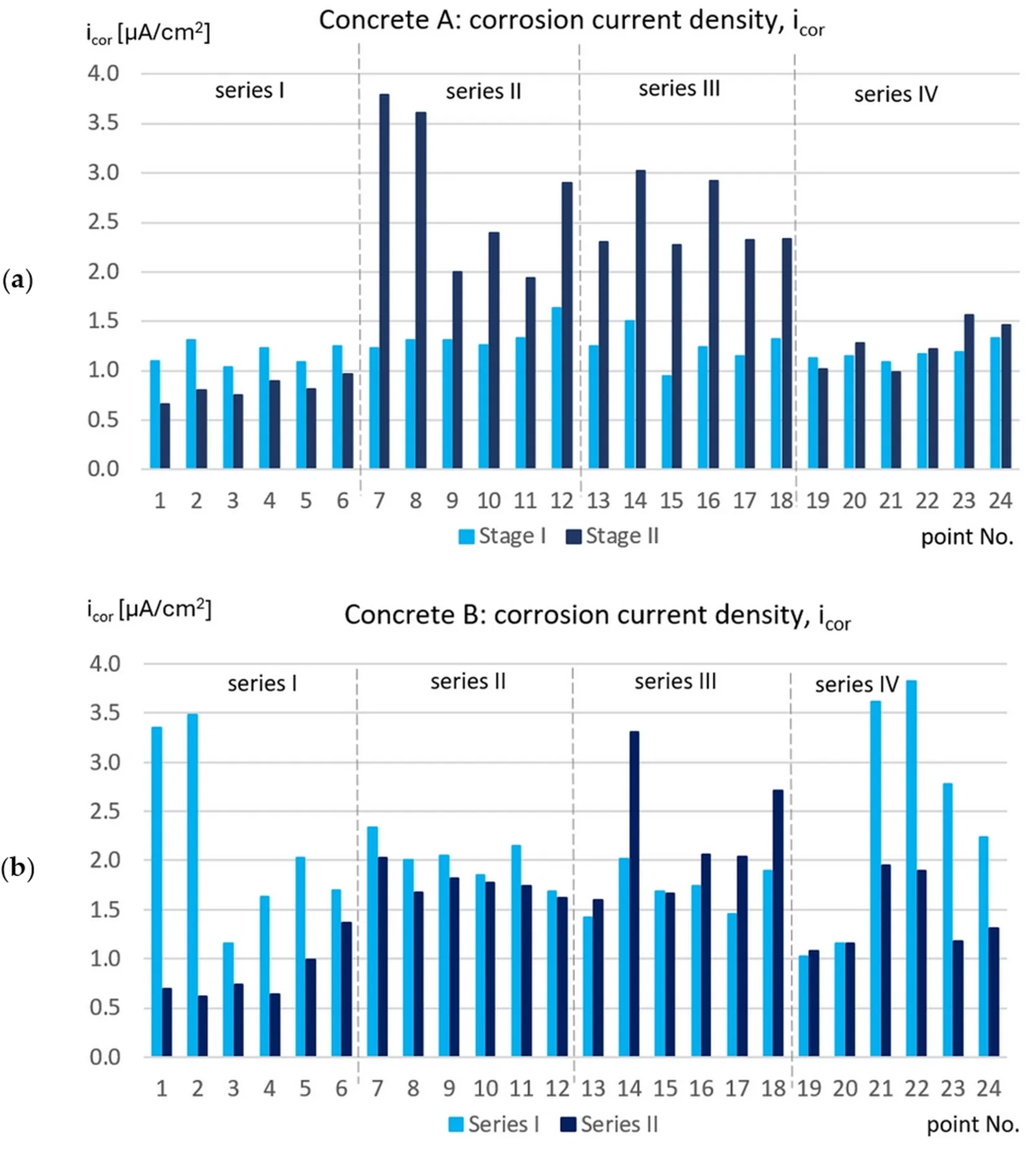
Changes in corrosion current density measured during the first and second measurement stages for: (**a**) Concrete A and (**b**) Concrete B.

**Figure 7:**
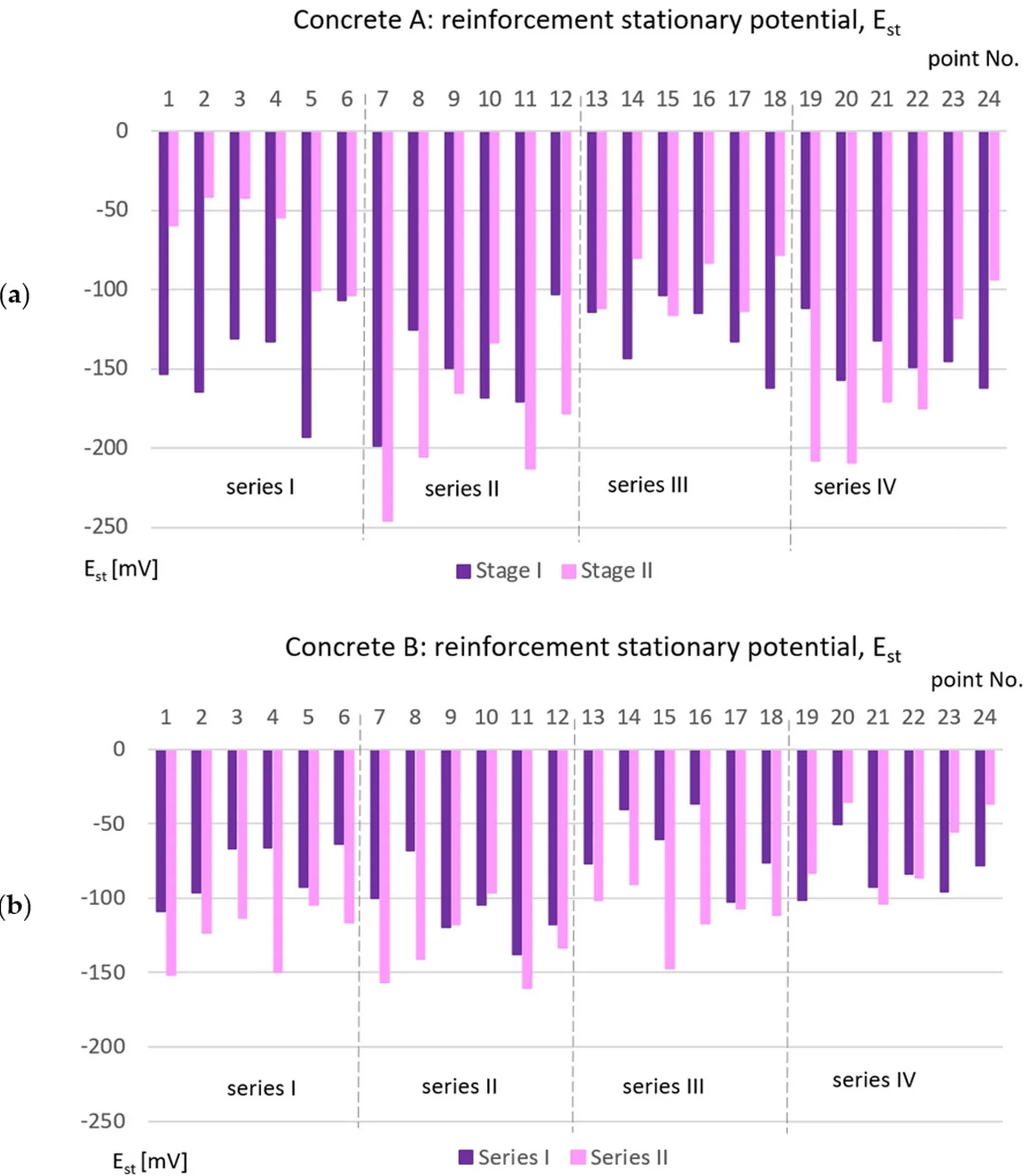
Changes in the stationary potential of the reinforcing steel measured during the first and second measurement stages for: (**a**) Concrete A and (**b**) Concrete B.

**Figure 8:**
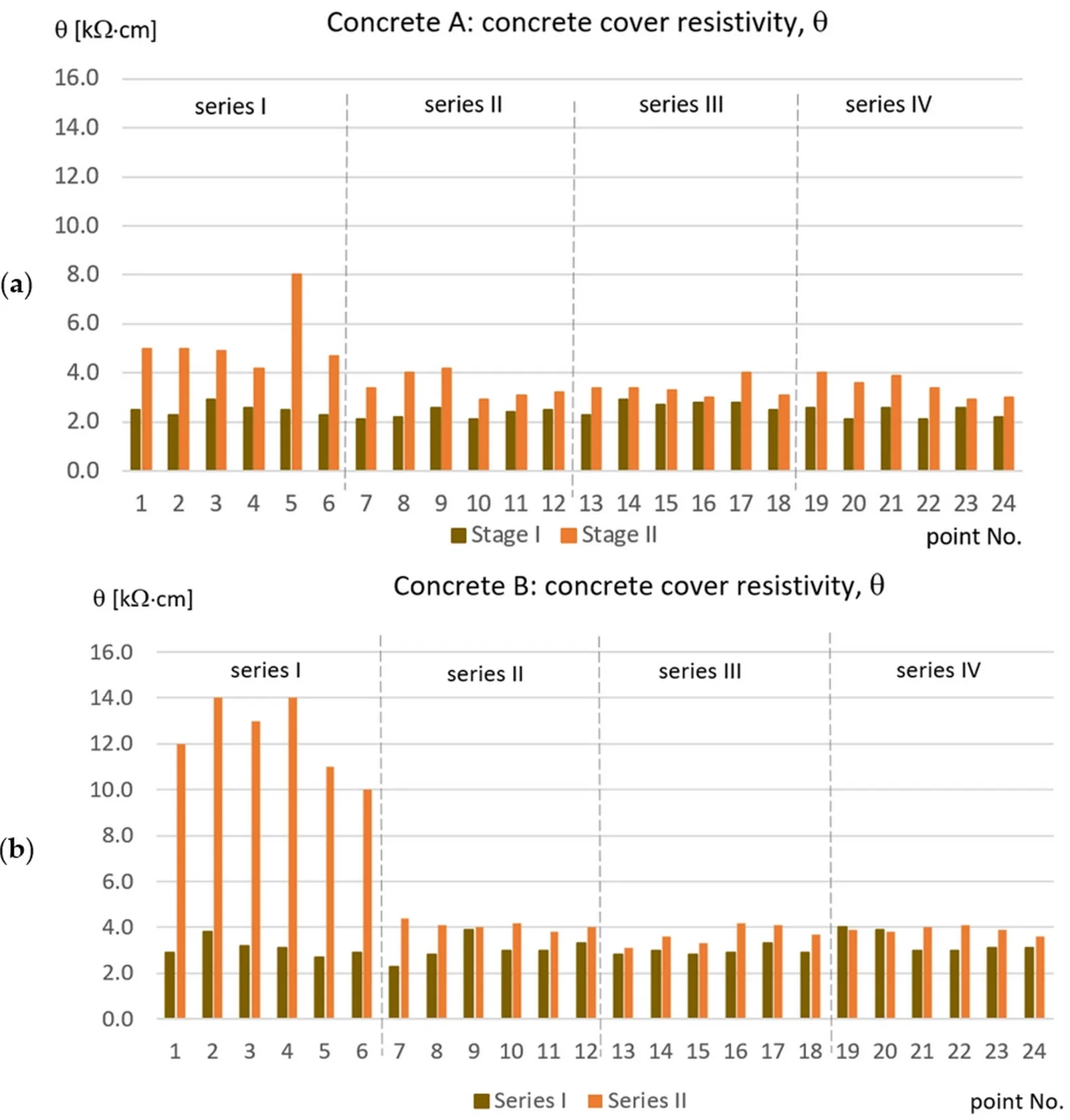
Changes in concrete cover resistivity measured during the first and second measurement stages for: (**a**) Concrete A and (**b**) Concrete B.

The results of the corrosion risk assessment of the reinforcing steel bars obtained using the Galvanostatic Pulse Method (GPM) are presented in Table 3 and Table 4 for Concrete A and Concrete B, respectively, and graphically illustrated in Figure 6, Figure 7 and Figure 8. The results of GPM measurements performed on Concrete A specimens were presented at the 11th WMCCAU conference in Ostrava [47]. Tables and figures present the results from measurements performed in two testing stages, with two measurements taken at designated locations on each specimen during each stage. The measured values were evaluated against the threshold criteria presented in Table 2. Three electrochemical parameters were determined: corrosion current density (i_cor_), stationary potential of the reinforcing steel (E_st_), and resistivity of the concrete cover (θ). Among these, the corrosion current density is the most informative parameter, as it directly reflects the corrosion activity of the reinforcing bar and allows the corrosion rate to be determined. The stationary potential and resistivity of the concrete cover allow only for an estimation of the probability of corrosion occurring near the measurement point.

#### 3.1.1. Analysis of Corrosion Current Density Results

A comparative analysis of the results of the corrosion current density revealed a pronounced influence of both the exposure conditions and the type of cement used on the corrosion activity of the reinforcing steel bars and the rate of corrosion (Table 3 and Table 4; Figure 6a,b). For all Concrete A specimens containing blast furnace cement, the corrosion current density measured during the first stage (reference measurements) ranged from i_cor_ = 0.95 ÷ 1.63 μA/cm^2^, indicating insignificant corrosion activity of the reinforcing steel (Table 2). In contrast, the corresponding values obtained for Concrete B were noticeably higher and exhibited greater variability, ranging from i_cor_ = 1.02 ÷ 3.82 μA/cm^2^. This difference can probably be attributed to the use of composite cement in Concrete B, which is characterized by a slower hydration and setting process. As a result, the concrete cover exhibited a lower electrical resistivity at the time of the test because cement hydration was still ongoing [48,49]. Consequently, the measured corrosion current density may overestimate the actual corrosion activity of the reinforcing steel, as the passive layer on the steel surface had not yet fully developed and the concrete cover had not yet reached sufficient electrical resistivity.

This interpretation is supported by the results obtained during the second measurement stage. In the Series I specimens, which were stored under laboratory conditions, the corrosion current density decreased to i_cor_ = 0.66 ÷ 0.97 μA/cm^2^ for Concrete A and i_cor_ = 0.62 ÷ 1.37 μA/cm^2^ for Concrete B. These values indicate a reduction in corrosion activity compared to the reference measurements, although the decrease was less pronounced for Concrete B.

In the Series II specimens, immersed in a 3% NaCl solution, Concrete A exhibited the greatest increase in the density of the corrosion current, with i_cor_ = 1.94 ÷ 3.79 μA/cm^2^. According to the assessment criteria (Table 2), these values correspond to low corrosion activity and an estimated corrosion rate of 0.022–0.044 mm/year. On the contrary, the corresponding values for Concrete B ranged from i_cor_ = 1.62 ÷ 2.03 μA/cm^2^ corresponding to a corrosion rate of 0.019 to 0.023 mm/year, and were even lower than those obtained during the reference measurements. These results indicate that the type of cement (blast furnace cement versus composite cement) had a substantial influence on the corrosion activity of the reinforcing steel under exposure to a 3% NaCl solution. The concrete produced with composite cement (Concrete B) provided considerably more effective protection against chloride-induced corrosion than the concrete containing blast furnace cement (Concrete A).

In Series III specimens, fully immersed in a 3% NaCl solution and simultaneously subjected to freeze–thaw cycling, the corrosion current density ranged from i_cor_ = 2.27 ÷ 3.02 μA/cm^2^ or Concrete A, corresponding to an estimated corrosion rate of 0.026–0.035 mm/year, and from i_cor_ = 1.60 ÷ 3.31 μA/for Concrete B, corresponding to a corrosion rate of 0.018–0.038 mm/year. Comparison of the results obtained for both concrete mixtures indicates that concrete containing composite cement was generally more effective in limiting the corrosion activity of the reinforcing steel under the combined action of chloride exposure and freeze–thaw cycling. With the exception of a single measurement point (i_cor_ = 3.31 μA/cm^2^) all values recorded for Concrete B were lower than those obtained for Concrete A.

In Series IV specimens, half-immersed in a 3% NaCl solution and subjected to freeze–thaw cycling, the corrosion current density of Concrete A remained almost unchanged, ranging from i_cor_ = 0.99 ÷ 1.56 μA/cm^2^. In contrast, Concrete B exhibited a slight decrease relative to the reference measurements, with i_cor_ = 1.08 ÷ 1.95 μA/cm^2^. One possible explanation for the obtained values is that, during the specimen exposure period, chloride ions may not yet have reached the reinforcement surface in concentrations sufficient to initiate corrosion.

However, given the absence of measurements regarding chloride concentrations and penetration profiles, this interpretation should be treated as a hypothesis requiring further experimental verification. Furthermore, the impact of microstructural damage caused by freeze–thaw cycles on the corrosion process in this instance cannot be conclusively determined.

#### 3.1.2. Analysis of Reinforcement Stationary Potential Results

The stationary potential values of the reinforcing steel obtained during the first measurement stage for both Concrete A and Concrete B did not exceed E_st_ = −200 mV at any of the measurement locations, indicating no significant risk of corrosion (Table 2), corresponding to an estimated probability of corrosion of approximately 5%.

During the second measurement stage, changes in the stationary potential were observed in the Concrete A specimens. In Series I, the stationary potential values increased compared to those recorded during the first stage (Figure 7a); however, they remained within the same range that corresponds to a minimal corrosion probability of approximately 5% (E_st_ > −200 mV). In Series II, three measurement locations exhibited stationary potential values below −200 mV (−246.10, −205.70, and −213.10 mV), indicating an increased corrosion probability of approximately 50% in these areas. These findings suggest that concrete containing blast furnace cement may provide insufficient protection against corrosion under exposure to a 3% NaCl solution.

In Series III and Series IV, in which the specimens were fully or partially immersed in a 3% NaCl solution and simultaneously subjected to freeze–thaw cycles, stationary potential results were inconclusive. Most measurement locations still indicated a low corrosion probability of approximately 5%; however, in two locations within the Series IV specimens, the measured stationary potentials were −208.20 and −209.18 mV, corresponding to an estimated corrosion probability of approximately 50%. In contrast, for all specimens produced with Concrete B (Figure 7b), regardless of the exposure conditions (Series I–IV), the stationary potential remained above E_st_ = −200 mV; consequently, the estimated corrosion probability remained at the minimum level of approximately 5% throughout the test program.

Overall, the stationary potential measurements did not allow unambiguous conclusions to be drawn, as they were not fully consistent with the results obtained from the corrosion current density and the concrete cover resistivity measurements. It should be noted, however, that the stationary potential is not the primary parameter for assessing reinforcement corrosion using the Galvanostatic Pulse Method (GPM) [50,51].

#### 3.1.3. Analysis of Concrete Cover Resistance Results

The concrete cover resistivity values obtained during the first measurement stage were very low for all specimens (Table 3 and Table 4; Figure 8a,b), ranging from Θ = 2.10–2.90 kΩ·cm for Concrete A and Θ = 2.30–3.90 kΩ·cm for Concrete B. According to the assessment criteria (Table 2), these values could incorrectly be interpreted as indicating a high probability of reinforcement corrosion (Θ < 10 kΩ·cm). However, in the present study, the low resistivity values were due to the early age of the specimens, their high moisture content, and the ongoing concrete curing and hydration processes [46,47]. During the second measurement stage, the resistivity of the concrete cover increased, most markedly in the Series I Concrete B specimens, where values ranged from Θ = 10.0–14.0 kΩ·cm, while a less pronounced increase was observed for Concrete A (Θ = 4.20–8.0 kΩ·cm). These results indicate that concrete specimens stored under laboratory conditions gradually developed higher electrical resistivity as the hydration process progressed. A moderate increase in the resistivity of the concrete cover was also observed in the remaining specimen series; however, the changes were considerably smaller. This was likely associated with the exposure conditions, including the presence of the 3% NaCl solution and repeated freeze–thaw cycles. In Series II, exposure to chloride ions may have slowed the activation of the blast furnace slag and the hydration of the clinker phases in both the blast furnace cement and the composite cement concretes compared to standard water curing [52]. In Series III and Series IV, the freeze–thaw cycles likely further inhibited the hydration process, thus limiting the increase in concrete cover resistivity.

### 3.2. Results of the Compressive Strength and Elasticity Modulus Tests

The compressive strength and modulus of elasticity of the concrete were determined based on tests performed on 150 × 150 × 150 mm cubic specimens and 300 × 150 mm cylindrical specimens, according to the relevant standards [41,42]. The results were used to determine the concrete strength class and the mean modulus of elasticity. A detailed summary of the test results is presented in Table 5.

**Figure 9:**
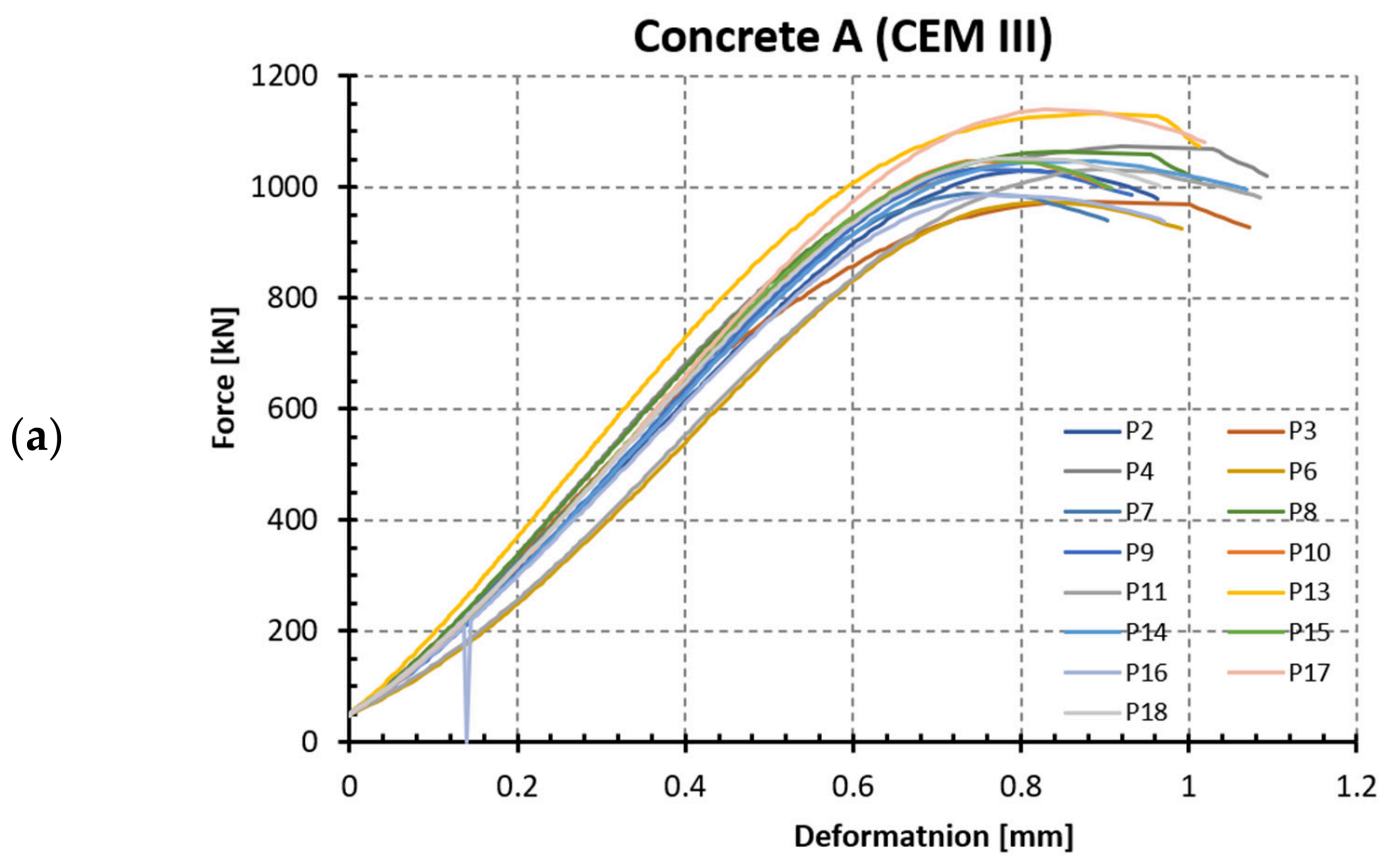

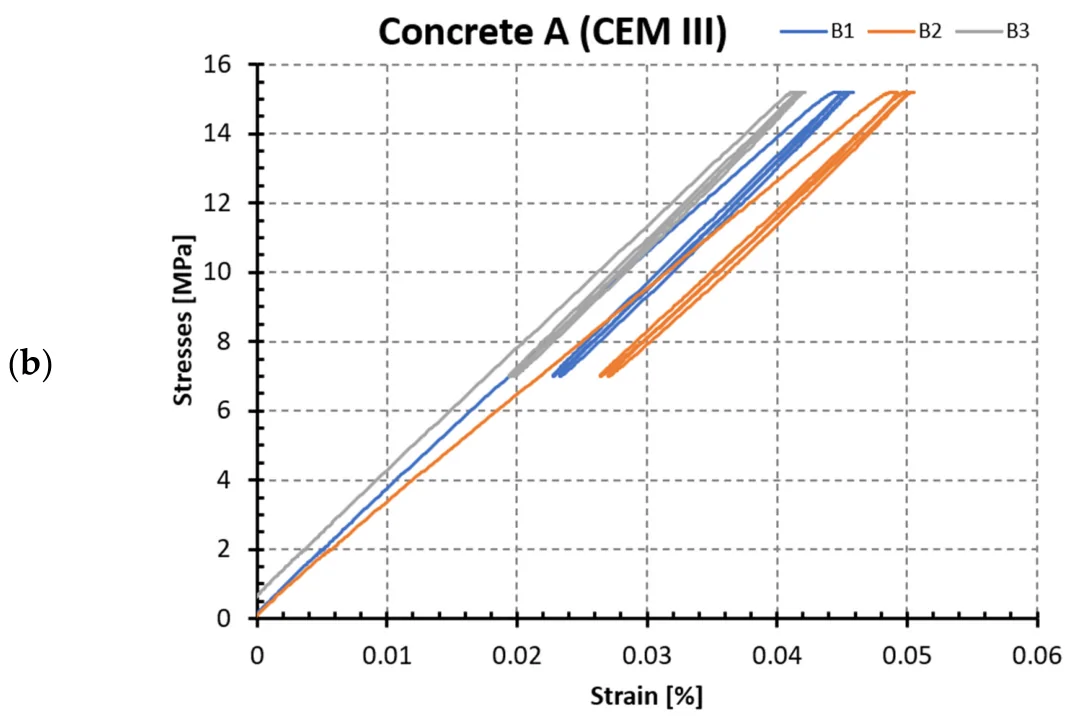
Representative curves for Concrete A: (**a**) load–displacement relationship and (**b**) stress–strain relationship used for determining the modulus of elasticity.

Based on the results obtained, Concrete A was classified as strength class C30/37, while Concrete B achieved strength class C35/45. The characteristic cube compressive strength of concrete was estimated in accordance with the conformity criteria specified in PN-EN 206:2014-04 [53]. Based on the test results, the maximum characteristic strength satisfying both conformity criteria was determined as the lower of the following two values: the minimum individual compressive strength increased by 4 MPa and the mean compressive strength reduced by 1.48 times the standard deviation. The corresponding concrete strength class was then assigned on the basis of the obtained characteristic cube compressive strength. The variability of the results was low for both concretes, with coefficients of variation ranging from 4.7 to 4.9% for compressive strength and from 3.1 to 6.5% for the modulus of elasticity. These low values indicate a high degree of homogeneity and excellent quality of the prepared concrete mixtures. Representative load–deformation curves (Figure 9a) and stress–strain curves used to determine the modulus of elasticity (Figure 9b) for Concrete A are presented in Figure 9.

### 3.3. Concrete Shrinkage Test Results

**Figure 10:**
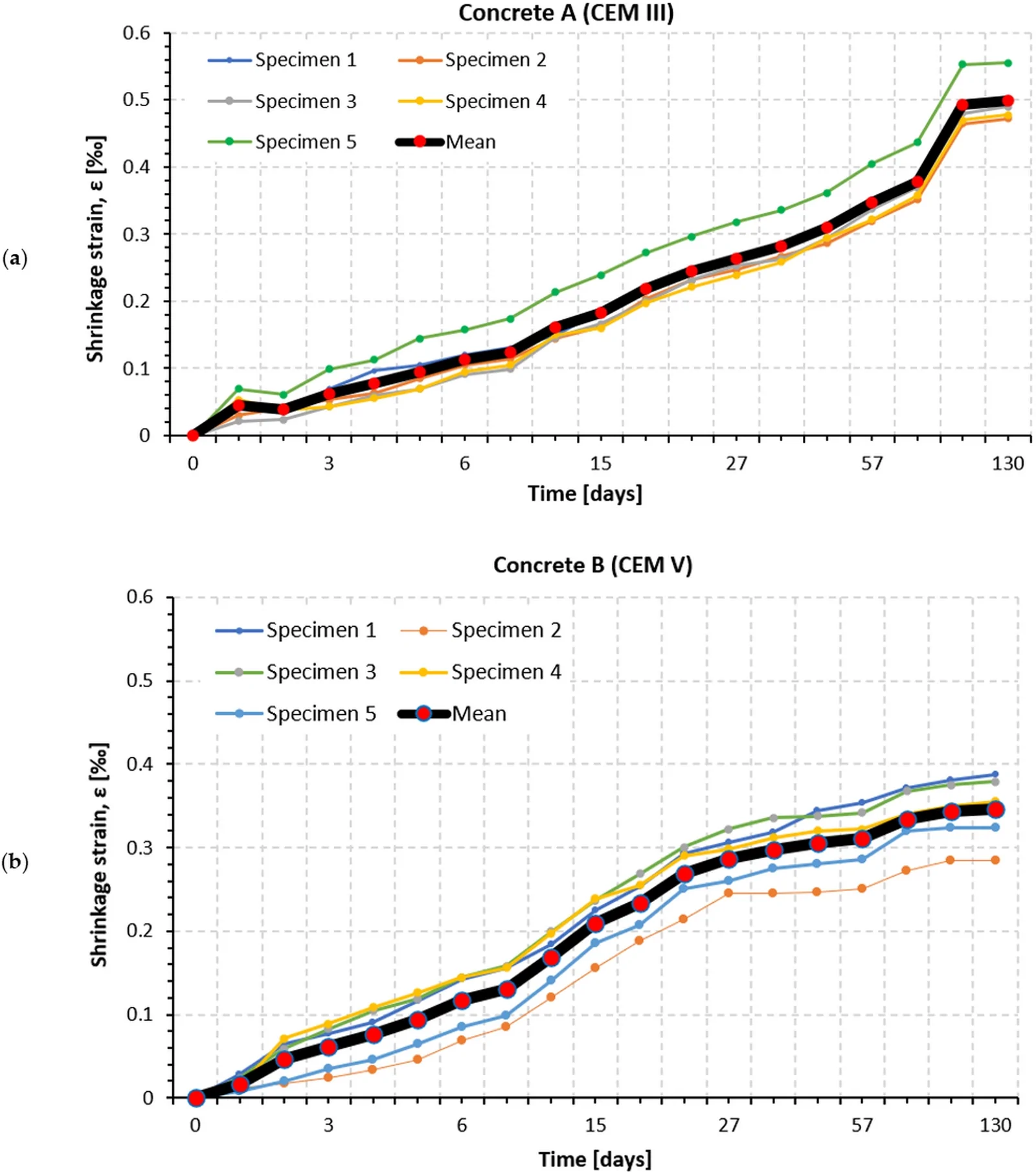
Mean shrinkage strain versus time: (**a**) measured for Concrete A specimens, (**b**) measured for Concrete B specimens.

Based on the measurements performed on all four faces of each specimen, the mean shrinkage strain was determined for each concrete type. The development of shrinkage strain is presented in Figure 10 for Concrete A (Figure 10a) and Concrete B (Figure 10b). The tests were carried out according to EN 12390-16:2019 [43].

A comparison of the results obtained for both concretes revealed distinct differences in the shrinkage process. Concrete B exhibited approximately 32% lower shrinkage strain at 130 days of measurement than Concrete A. Furthermore, a tendency toward earlier stabilization of shrinkage strains was observed in Concrete B. The lower shrinkage observed in the concrete containing CEM V may be related to the presence of fly ash, which, according to previous studies, can contribute to the refinement of the pore structure and reduction in drying shrinkage [54,55]. However, since no SEM, MIP, or porosity measurements were performed in the present study, this mechanism cannot be directly confirmed for the investigated concrete. It was also observed that the temperature and relative humidity fluctuations recorded during the tests (Figure 4), including short-term decreases in relative humidity, may have temporarily affected the shrinkage strain development. However, no abrupt changes in the shrinkage curves corresponding to these fluctuations were observed, and the overall shrinkage trend was not noticeably disturbed (Figure 10a,b).

### 3.4. Freeze–Thaw Resistance Test Results

The freeze–thaw resistance test was performed using the standard method according to PN-B-06265:2022-08 (Annex N) [45], in which the specimens were subjected to repeated freeze–thaw cycles under fully water-saturated conditions. The freezing—thaw resistance of the concrete was evaluated based on changes in compressive strength, determined by the uniaxial compression test, and mass loss after completion of the freeze–thaw cycles. The test results are summarized in Table 6 (Concrete A) and Table 7 (Concrete B). Based on the obtained results, it can be concluded that after 300 freeze–thaw cycles, only a slight reduction in compressive strength was observed, amounting to 10.4% for Concrete A and 6.9% for Concrete B. In both cases, the reduction remained below the 20% limit specified in the standard. Similarly, the mass loss of the specimens was 0.40% for Concrete A and 0.24% for Concrete B, well below the permissible limit of 5%. These findings indicate that both concretes exhibited good freeze–thaw resistance, with Concrete B demonstrating superior performance.

### 3.5. Water Absorption Test Results

The results of the water absorption test are presented in Table 8. Based on these results, the mean water absorption of Concrete A was 5.96%, while that of Concrete B was 5.06%. Therefore, both concretes exceeded the 5% limit specified in PN-B-06250 [46], although for Concrete B the exceedance was only 0.06 percentage points. Water absorption was treated in this study as an additional parameter characterizing the investigated concretes rather than as an acceptance criterion for their inclusion in the experimental program. Moreover, no clear deterioration in the corrosion-related measurements corresponding to the increased water absorption was observed during the investigated period.

## 4. Conclusions

Based on experimental studies, which included an assessment of reinforcement corrosion rates and selected mechanical and material properties of concretes made with two types of cement (CEM III and CEM V) under conditions of chloride exposure and the combined action of chlorides and freeze–thaw cycles, the following observations and conclusions were formulated:

1. The type of cement used in the concrete, specifically blast furnace cement (CEM III) or composite cement (CEM V), influences the durability of reinforced concrete, particularly regarding the protective role of the concrete cover against reinforcement corrosion.
2. The corrosion current density, determined by the galvanostatic pulse method (GPM), was generally lower for reinforcing steel embedded in Concrete B (CEM V) than in Concrete A (CEM III).
3. Measurements of the stationary potential of the reinforcement, performed using the GPM method, indicated a lower probability of corrosion development in specimens made of concrete with CEM V cement.
4. Concrete cover resistivity measurements performed on relatively young specimens should be interpreted with caution. At this stage, the results may be affected by the high moisture content and ongoing maturation of the concrete, which can influence the electrochemical response.
5. Replacing CEM III with CEM V, while maintaining the remaining mixture proportions, resulted in higher compressive strength for the investigated concrete and, consequently, in a higher strength class.
6. Analysis of the shrinkage test results revealed that, after 130 days of measurements, the average final shrinkage value was approximately 32% lower in concrete made with CEM V cement than in concrete made with CEM III cement.
7. Both concretes showed satisfactory behaviour during the freeze–thaw tests under the conditions adopted in this study, with Concrete B showing a smaller reduction in compressive strength after cycling. The water absorption of Concrete B (5.06%) was lower than that of Concrete A (5.96%); however, both values slightly exceeded the adopted 5% limit.

It should be noted that the conclusions presented in this study are based on a limited number of specimens and therefore require further verification. The present study constitutes the first stage of a long-term research program aimed at assessing the durability of reinforced concrete elements under various environmental conditions.

## Figures and Tables

**Table 1:** Mix proportions of the concrete mixtures.

	Components	Content [kg/m^3^]
Cement (optionally)	Concrete A: Cement CEM III/A 42.5 N MSR NA/	384
	Concrete B: Cement CEM V/A (S-V) 42.5N-LH/HSR/NA	
Aggregate	Natural sand 0–2 mm	680
	Basalt aggregate 2–8 mm	600
	Basalt aggregate 8–16 mm	650
Water	Tap water	166
Admixtures	Superplasticizer MasterGlenium ACE 430 TR	9.92
	Air-entraining admixture MasterAir 107	1.15
w/c ratio = 0.43		

**Table 2:** Criteria for assessing the corrosion risk of reinforcing steel in concrete using the Galvanostatic Pulse Method (GPM) [40].

Corrosion current density, i_cor_ [μ A/cm^2^]	Corrosion activity of reinforcing steel		Predicted corrosion rate
	<0.5	No corrosion predicted	<0.006 mm/year
	0.5 ÷ 2.0	Insignificant	0.006 ÷ 0.023 mm/year
	2.0 ÷ 5.0	low	0.023 ÷ 0.058 mm/year
	5.0 ÷ 15.0	moderate	0.058 ÷ 0.174 mm/year
	>15.0	high	>0.174 mm/year
Concrete cover resistivity, Θ [K Ω· cm]	≥20	Low probability of corrosion	
	10 ÷ 20	Moderate probability of corrosion	
	≤10	High probability of corrosion	
Stationary potential of reinforcing steel, E_st_ [mV]	>−200	5% probability of corrosion	
	−350 ÷ −200	50% probability of corrosion	
	<−350	95% probability of corrosion	

**Table 3:** Results of the Galvanostatic Pulse Method (GPM) measurements for Concrete A specimens.

Concrete A: Specimens Made of Concrete with CEM III Blast Furnace Cement								
Specimen			Parameter					
			Corrosion Current Density, i_cor_		Reinforcement Stationary Potential, E_st_		Concrete Cover Resistivity, θ	
Type	No.	Point No.	Stage I	Stage II	Stage I	Stage II	Stage I	Stage II
Series I (refer.)	1	1_1	1.10	0.66	−153.27	−59.44	2.50	5.00
		2_1	1.31	0.80	−164.58	−41.65	2.30	5.00
	2	1_1	1.04	0.75	−130.81	−41.89	2.90	4.90
		2_1	1.23	0.90	−132.99	−54.53	2.60	4.20
	3	1_1	1.09	0.81	−193.28	−100.63	2.50	8.00
		2_1	1.25	0.97	−106.47	−103.51	2.30	4.70
Series II	4	1_1	1.23	3.79	−192.41	−246.10	2.10	3.40
		2_1	1.31	3.61	−125.19	−205.70	2.20	4.00
	5	1_1	1.31	2.00	−149.29	−164.90	2.60	4.20
		2_1	1.26	2.39	−168.33	−133.30	2.10	2.90
	6	1_1	1.33	1.94	−170.35	−213.10	2.40	3.10
		2_1	1.63	2.90	−102.72	−177.80	2.50	3.20
Series III	7	1_1	1.25	2.30	−114.27	−111.60	2.30	3.40
		2_1	1.50	3.02	−143.06	−79.79	2.90	3.40
	8	1_1	0.95	2.27	−103.89	−116.00	2.70	3.30
		2_1	1.24	2.92	−114.74	−83.30	2.80	3.00
	9	1_1	1.15	2.32	−132.91	−113.40	2.80	4.00
		2_1	1.32	2.33	−162.24	−77.92	2.50	3.10
Series IV	10	1_1	1.13	1.02	−111.38	−208.20	2.60	4.00
		2_1	1.15	1.28	−156.86	−209.18	2.10	3.60
	11	1_1	1.09	0.99	−132.27	−170.43	2.60	3.90
		2_1	1.17	1.22	−148.77	−175.20	2.10	3.40
	12	1_1	1.19	1.56	−145.31	−117.90	2.60	2.90
		2_1	1.33	1.46	−161.77	−93.84	2.20	3.00

**Table 4:** Results of the Galvanostatic Pulse Method (GPM) measurements for Concrete B specimens.

Concrete B: Specimens Made of Concrete with CEM V								
Specimen			Parameter					
			Corrosion Current Density, i_cor_		Reinforcement Stationary Potential, E_st_		Concrete Cover Resistivity, θ	
Type	No.	Point No.	Stage I	Stage II	Stage I	Stage II	Stage I	Stage II
Series I (refer.)	1	1_1	3.35	0.69	−109.00	−151.70	2.90	12.00
		2_1	3.48	0.62	−96.15	−123.40	3.80	14.00
	2	1_1	1.16	0.74	−66.90	−113.10	3.20	13.00
		2_1	1.63	0.64	−65.97	−150.06	3.10	14.00
	3	1_1	2.03	0.99	−92.64	−104.67	2.70	11.00
		2_1	1.70	1.37	−63.86	−116.56	2.90	10.00
Series II	4	1_1	2.34	2.03	−99.89	−156.90	2.30	4.40
		2_1	2.00	1.68	−68.07	−141.00	2.80	4.10
	5	1_1	2.05	1.82	−119.70	−117.90	3.90	4.00
		2_1	1.85	1.77	−104.50	−96.62	3.00	4.20
	6	1_1	2.15	1.74	−137.70	−160.70	3.00	3.80
		2_1	1.68	1.62	−118.03	−133.30	3.30	4.00
Series III	7	1_1	1.42	1.60	−76.96	−101.30	2.80	3.10
		2_1	2.02	3.31	−40.70	−90.77	3.00	3.60
	8	1_1	1.68	1.67	−60.82	−147.10	2.80	3.30
		2_1	1.74	2.06	−36.73	−116.90	2.90	4.20
	9	1_1	1.45	2.04	−102.70	−107.30	3.30	4.10
		2_1	1.89	2.71	−76.03	−111.80	2.90	3.70
Series IV	10	1_1	1.02	1.08	−101.70	−83.28	4.00	3.90
		2_1	1.16	1.15	−50.76	−35.79	3.90	3.80
	11	1_1	3.61	1.95	−92.41	−103.80	3.00	4.00
		2_1	3.82	1.89	−83.75	−86.32	3.00	4.10
	12	1_1	2.78	1.18	−95.91	−55.91	3.10	3.90
		2_1	2.24	1.31	−77.90	−36.73	3.10	3.60

**Table 5:** Compressive strength and Young’s modulus of Concretes A and B.

** **	f_cm,cube_ [MPa]	f_ck,cube_ [MPa]	s [MPa]	ν [%]	E_cm_ [GPa]	s [GPa]	ν [%]
Concrete A	46.3	42.3	2.20	4.9	33.1	2.2	6.5
Concrete B	53.7	49.7	2.20	4.7	43.7	1.4	3.1

**Table 6:** Compressive strength and mass loss of Concretes A and B after 300 freeze–thaw cycles (concrete A).

Concrete A						
Reference Specimens		Specimens Subjected to Freeze–Thaw Cycles				
Specimen No.	f_ci,o_ [MPa]	Specimen No.	f_ci,m_ [MPa]	M_1_ [g]	M_2_ [g]	$∆M=\frac{M_{1}-M_{2}}{M_{1}}$[%]
P1	55.03	M1 CEM III	53.99	2444	2433	0.45
P2	56.76	M2 CEM III	49.43	2498	2488	0.40
P3	58.59	M3 CEM III	49.28	2413	2405	0.33
f_cm,o_ [MPa]	56.80	f_cm,m_ [MPa]	50.90	Mean percentage mass loss of the specimens $∆M_{m}[\%]$		0.40 ≤ 5%
Mean percentage reduction in compressive strength of the tested specimens $∆f_{cm}=\frac{f_{cm,o}-f_{cm,m}}{f_{cm,o}}[\%]$			10.4% ≤ 20%			

f_ci,o_—compressive strength of the *i*-th reference specimen. f_ci,m_—compressive strength of the *i*-th specimen subjected to freeze–thaw cycles. f_cm,o_—mean compressive strength of the reference specimens. M_1_—mass of the specimen before freeze–thaw cycle. M_2_—mass of the specimen after freeze–thaw cycle. ΔM—percentage mass loss of the specimens subjected to freeze–thaw cycle.

**Table 7:** Compressive strength and mass loss of Concretes A and B after 300 freeze–thaw cycles (concrete B).

Concrete B						
Reference Specimens		Specimens Subjected to Freeze–Thaw Cycles				
Specimen No.	f_ci,o_ [MPa]	Specimen No.	f_ci,m_ [MPa]	M_1_ [g]	M_2_ [g]	$∆M=\frac{M_{1}-M_{2}}{M_{1}}$[%]
Por 1	62.25	Mo1 CEM V	58.08	2482.2	2478.0	0.17
Por 2	62.94	Mo2 CEM V	54.96	2420.9	2416.4	0.19
Por 3	65.46	Mo3 CEM V	61.84	2484.8	2474.8	0.40
Por 4	62.85	Mo4 CEM V	61.81	2466.0	2460.9	0.21
Por 5	65.69	Mo5 CEM V	61.02	2469.8	2462.5	0.30
Por 6	64.89	Mo6 CEM V	59.86	2453.4	2449.1	0.18
f_cm,o_ [MPa]	64.01	f_cm,m_ [MPa]	59.60	Mean percentage mass loss of the specimens $∆M_{m}[\%]$		0.24 ≤ 5%
Mean percentage reduction in compressive strength of the tested $∆f_{cm}=\frac{f_{cm,o}-f_{cm,m}}{f_{cm,o}}[\%]$			6.90% ≤ 20%			

f_ci,o_—compressive strength of the *i*-th reference specimen. f_ci,m_—compressive strength of the *i*-th specimen subjected to freeze–thaw cycles. f_cm,o_—mean compressive strength of the reference specimens. M_1_—mass of the specimen before freeze–thaw cycle. M_2_—mass of the specimen after freeze–thaw cycle. ΔM—percentage mass loss of the specimens subjected to freeze–thaw cycle.

**Table 8:** Mass of water-saturated and oven-dried specimens for Concretes A and B.

No	M_m_ [g]	M_s_ [g]	${∆M=M}_{m}-M_{s}$ [g]	$N=\frac{∆M}{M_{s}}\cdot 100\%[\%]$
Concrete A				
NS1 CEM III	2442.2	2302.6	139.6	6.06
NS2 CEM III	2475.4	2330.6	144.8	6.21
NS3 CEM III	2452.1	2321.8	130.3	5.61
			N_av,CEM III_ [%]	5.96
			s_CEM I_	0.25
			ν_CEM II_ [%]	4.28
Concrete B				
NS1 CEM V	2474.4	2356.9	117.5	4.99
NS2 CEM V	2468.6	2347.6	121.0	5.15
NS3 CEM V	2455.1	2337.1	118.0	5.05
			N_av,CEM V_ [%]	5.06
			s_CEM V_	0.25
			ν_CEM V_ [%]	1.30

M_m_—mass of the water-saturated specimen. M_s_—mass of the oven-dried specimen. ΔM—difference between the mass of the water-saturated and oven-dried specimen. N—water absorption. N_av_—mean water absorption.

## Data Availability

The original contributions presented in this study are included in the article. Further inquiries can be directed to the corresponding author.

## References

[1] Alaux N., Bechstedt N., Zhong X., Mastrucci A., Ramon D., Steinberger-Maierhofer D., Allacker K., Passer A., Röck M. (2026). Context-specific life cycle emissions pathways for EU buildings and construction. Nat. Commun..

[2] Cavalett O., Watanabe M.D.B., Voldsund M., Roussanaly S., Cherubini F. (2024). Paving the way for sustainable decarbonization of the European cement industry. Nat. Sustain..

[3] Shah I.H., Miller S.A., Jiang D., Myers R.J. (2022). Cement substitution with secondary materials can reduce annual global CO_2_ emissions by up to 1.3 gigatons. Nat. Commun..

[4] Snellings R., Suraneni P., Skibsted J. (2023). Future and emerging supplementary cementitious materials. Cem. Concr. Res..

[5] Ahmad M.R., Fernàndez-Jimenez A., Chen B., Leng Z., Dai J.-G. (2024). Low-carbon cementitious materials: Scale-up potential, environmental impact and barriers. Constr. Build. Mater..

[6] Akintayo B.D., Babatunde O.M., Akintayo D.C., Olanrewaju O.A. (2025). Transforming Industrial Waste into Low-Carbon Cement: A Multi-Criteria Assessment of Supplementary Cementitious Materials for Sustainable Concrete Design. Recycling.

[7] Albuhairi D., Di Sarno L. (2022). Low-Carbon Self-Healing Concrete: State-of-the-Art, Challenges and Opportunities. Buildings.

[8] Wandoch K., Tałaj M. (2021). New Cement CEM II/C-M and CEM VI in Frost-Resistant and High-Strength Concrete. Mater. Bud..

[9] Król A., Giergiczny Z., Kuterasińska-Warwas J. (2020). Properties of Concrete Made with Low-Emission Cements CEM II/C-M and CEM VI. Materials.

[10] Jaskulski R., Jóźwiak-Niedźwiedzka D., Yakymechko Y. (2020). Calcined Clay as Supplementary Cementitious Material. Materials.

[11] Gołaszewska M., Giergiczny Z. (2021). Study of the Properties of Blended Cements Containing Various Types of Slag Cements and Limestone Powder. Materials.

[12] Jin H., Ghazizadeh S., Provis J.L. (2024). Thermodynamic modelling of alkali-silica reactions in blended cements. Cem. Concr. Res..

[13] Da Silva P.R., de Brito J. (2015). Experimental study of the porosity and microstructure of self-compacting concrete (SCC) with binary and ternary mixes of fly ash and limestone filler. Constr. Build. Mater..

[14] Pellerano M., Samson G., Patapy C., Calviac A., Cyr M. (2024). Development of self-compacting low-carbon concretes for precast industry. Constr. Build. Mater..

[15] von Greve-Dierfeld S., Lothenbach B., Vollpracht A., Wu B., Huet B., Andrade C., Medina C., Thiel C., Gruyaert E., Vanoutrive H. (2020). Understanding the carbonation of concrete with supplementary cementitious materials: A critical review by RILEM TC 281-CCC. Mater. Struct..

[16] Dong B., Zhao S., Zhang Y., Zhang Y., Kitipornchai S., Yang J. (2026). Chloride Ingress Mechanisms-Informed Virtual Reliability Assessment of Corroded Reinforced Concrete Structures. Struct. Saf..

[17] Dong B., Yu Y., Feng Y., Wu D., Zhao G., Liu A., Gao W. (2023). Robust Numerical Solution for Assessing Corrosion of Reinforced Concrete Structures under External Power Supply. Eng. Struct..

[18] (2004). Eurocode 2: Design of Concrete Structures—Part 1-1: General Rules and Rules for Buildings.

[19] Phung Q.T., Frederickx L., Nguyen T.N., Nguyen V.T. (2023). Resistance of ordinary and low-carbon cements to carbonation: Microstructural and mineralogical alteration. Cem. Concr. Compos..

[20] Lollini F., Redaelli E. (2021). Carbonation of blended cement concretes after 12 years of natural exposure. Constr. Build. Mater..

[21] Irico S., Qvaeschning D., Mutke S., Deuse T., Gastaldi D., Canonico F. (2021). Durability of high performance self-compacting concrete with granulometrically optimized slag cement. Constr. Build. Mater..

[22] Tahwia A.M., Elgendy G.M., Amin M. (2021). Durability and microstructure of eco-efficient ultra-high-performance concreto. Constr. Build. Mater..

[23] Thummar C., Kondraivendhan B., Modhera C. (2024). Review on Rebar Corrosion in Alkali-Activated Concrete Subjected to Chloride-Rich Environment. IOP Conf. Ser. Earth Environ. Sci..

[24] Revert A.B., Danner T., Geiker M.R. (2024). Carbonation and corrosion of steel in fly ash concrete, concluding investigation of five-year-old laboratory specimens and preliminary field data. Cement.

[25] Achenbach R., Raupach M. (2024). Passivation of Steel Reinforcement in Low Carbon Concrete. Buildings.

[26] Tałaj M., Batog M., Jaśniok T., Giergiczny Z. (2024). Assessment of the Protective Potential of Reinforcing Steel in Low Carbon Footprint Concrete Using Electrical Resistance Corrosion Monitoring. Ochr. Przed Koroz..

[27] Sirivivatnanon V., Xue C., Khatri R. (2023). Long-term reinforcement corrosion in low carbon concrete with a high volume of SCMs exposed to NaCl solutions and field marine environment. Constr. Build. Mater..

[28] Akinwale A.E. (2018). The Effect of South African Quaternary Supplementary Cementitious Blends on Corrosion Behaviour of Concrete Reinforcement in Chloride and Sulphate Media. Ph.D. Thesis.

[29] Baltazar-Zamora M.A., Landa-Ruiz L., Landa-Gómez A., Santiago-Hurtado G., Moreno-Landeros V., Ramírez C.T.M., Rosales V.F., Croche R. (2024). Corrosion of AISI 316 Stainless Steel Embedded in Green Concrete with Low Volume of Sugar Cane Bagasse Ash and Silica Fume exposed in Seawater. Eur. J. Eng. Technoilogy Res..

[30] Baltazar-García B.P., Baltazar-Zamora D.F., Landa-Ruiz L., Méndez C.T., Solorzano R., Reyes J., Márquez S., Santiago-Hurtado G., Moreno-Landeros V., Croche R. (2022). Corrosion Behavior of AISI 1018 Reinforcing Steel in Sustainable Concrete Made with Sugar Cane Bagasse Ash and Recycled Aggregates Exposed in Seawater. Eur. J. Eng. Technol. Res..

[31] Babaee M., Castel A. (2016). Chloride-induced corrosion of reinforcement in low-calcium fly ash-based geopolymer concreto. Cem. Concr. Res..

[32] Badar S., Kupwade-Patil K., Bernal S.A., Provis J.L., Allouche E.N. (2014). Corrosion of steel bars induced by accelerated carbonation in low and high calcium fly ash geopolymer concretes. Constr. Build. Mater..

[33] Zhu J., Li Z., Huang Y., Li Y., Huang Y., Min H. (2025). Experimental Study on the Coupling of Freeze-Thaw Cycle and Chloride Corrosion of Alkali Slag Cementitious Materials. Polymers.

[34] Kessler S., Thiel C., Grossego C.U., Gehlena C. (2017). Effect of Freeze–Thaw Damage on Chloride Ingress into Concrete. Mater. Struct..

[35] Qian S., Baldock B., Qu D., Bouzoubaâ N., Gu P., Fournier B. Corrosion Performance of Reinforcing Steel in Concrete Containing Supplementary Cementing Materials. Proceedings of the NACE Northern Area Conference.

[36] (2019). Testing Fresh Concrete—Part 2: Slump-Test.

[37] (2019). Testing Fresh Concrete—Part 7: Air Content—Pressure Methods.

[38] (2019). Testing Hardened Concrete—Part 2: Making and Curing Specimens for Strength Tests.

[39] Raczkiewicz W., Wójcicki A. (2020). Temperature Impact on the Assessment of Reinforcement Corrosion Risk in Concrete by Galvanostatic Pulse Method. Appl. Sci..

[40] https://www.germanninstruments.com/wp-content/uploads/2022/02/GalvaPulse-GP-TDS-01.pdf.

[41] (2019). Testing Hardened Concrete—Part 3: Compressive Strength of Test Specimens.

[42] (2021). Testing Hardened Concrete—Part 13: Determination of Secant Modulus of Elasticity in Compression.

[43] (2020). Testing Hardened Concrete—Part 16: Determination of the Shrinkage of Concrete.

[44] (1998). Testing the Mechanical Properties of Concrete on Specimens Cast in Moulds.

[45] (2021). Concrete—Requirements, Performance, Production and Conformity—National Supplement to PN-EN 206+A2:2021-8.

[46] (1988). Ordinary Concrete.

[47] Raczkiewicz W., Bacharz K., Grajek A. Assessment of the Impact of Chlorides and Freeze-Thaw Cycles on the Reinforcement Corrosion Process in Concrete With Blast-Furnace Slag Cement. Proceedings of the 11th World Multidisciplinary Congress on Civil Engineering, Architecture and Urban Planning.

[48] McCafferty E. (2005). Validation of corrosion rates measured by the Tafel extrapolation method. Corros. Sci..

[49] Schneck U. (2019). Insights from corrosion current measurements on corrosion mechanisms in reinforced concrete and on the evaluation of other corrosion data. MATEC Web Conf..

[50] Sørensen H.E., Frølund F. Monitoring of reinforcement corrosion in marine concrete structures by the galvanostatic pulse method. Proceedings of the International IABSE Conference on Concrete in Marine Environments.

[51] Verma S.K., Bhadauria S.S., Akhtar S. (2014). Monitoring Corrosion of Steel Bars in Reinforced Concrete Structures. Sci. World J..

[52] Polder R.B., Peelen W.H.A. (2002). Characterization of chloride transport and reinforcement corrosion in concrete under cyclic wetting and drying by electrical resistivity. Cem. Concr. Compos..

[53] (2014). Concrete—Specification, Performance, Production and Conformity.

[54] Hu X., Shi Z., Shi C., Wu Z., Tong B., Ou Z., de Schutter G. (2017). Drying Shrinkage and Cracking Resistance of Concrete Made with Ternary Cementitious Components. Constr. Build. Mater..

[55] Segawa M., Kurihara R., Aili A., Igarashi G., Maruyama I. (2024). Shrinkage Reduction Mechanism of Low Ca/Si Ratio C-A-S-H in Cement Pastes Containing Fly Ash. Cem. Concr. Res..

